# Psychometric Properties of the Moore Index of Nutrition Self-Care in Arabic: A Study among Saudi Adolescents at King Saud University, Riyadh, Saudi Arabia

**DOI:** 10.1155/2020/9809456

**Published:** 2020-04-08

**Authors:** Adel Bashatah, Khalid A. Alahmary

**Affiliations:** ^1^Department of Administration and Education, College of Nursing, King Saud University, Riyadh, Saudi Arabia; ^2^College of Public Health King Saud Bin Abdul-Aziz University for Health Science, Riyadh, Saudi Arabia

## Abstract

**Method:**

The psychometric characteristics of MIN-SC were assessed using college freshman students at King Saud University in Riyadh, Saudi Arabia. The validity and reliability were examined using Cronbach's alpha coefficient. The construct validity was examined through principal component analysis.

**Results:**

The MIN-SC instrument was shown to be internally consistent with reliable scoring (Cronbach's alpha = 0.910). Exploratory factor analysis resulted in 42 items loading on three main components: estimative, production, and transitional, with a factor loading of eigenvalues >2. The final model explained 38% of the variance.

**Conclusion:**

The Arabic version of MIN-SC was shown to be a valid and reliable tool for assessing attitude toward nutrition among adolescent students.

## 1. Introduction

It is vital for adolescents to practice well-balanced nutrition. This is particularly important for school students who are still in their development stages [1, 2]. Nutrition may significantly affect student performance in both physical and mental applications, including those related to performance in the educational setting [1–3]. Adequate dietary intake is thus regarded as a fundamental factor for delivering high-quality health care to adolescents.

Here, self-care practices are important. This include healthy eating habits, sufficient physical activity, proper nutritional intake, risk reduction, and healthy coping habits [4]. Individuals should also monitor their weight and perform activities designed to manage the symptoms of unnecessary weight gain and obesity. Previous studies have reported that adequate self-care practices can recover metabolic control [5] and help individuals regain their quality of life [6]. These activities also reduce the risk of chronic diseases and disease-related complications [7].

Rapid economic changes in Saudi Arabia and many other countries are increasingly contributing to chronic diseases resulting from obesity and weight gain [8–10]. The World Health Organization (WHO) has reported that over 39% of adults are overweight and 14% are obese worldwide, with nearly 41 million children suffering from these problems [11]. In Saudi Arabia, the overall prevalence of obesity is 52.9% for both genders; this number may reach 59.5% by 2022 [12]. Factors contributing to these chronic conditions include lack of physical activity, consumption of high-fat foods, and behavioral and environmental changes [13].

Consistent with the Saudi Vision 2030, the Saudi Food and Drug Authority (SFDA) has launched its Strategy of Regulating Healthy Food Habits, which aims to encourage adequate caloric and nutritional intake by decreasing the levels of dietary sugar, salt, and saturated and trans fats in food products. The strategy also raises awareness and urges food product manufacturers and importers to reduce these contents. Meanwhile, restaurants and cafes are encouraged to include caloric information on menus so that consumers can more easily determine their daily intake. Efforts also include a nationwide survey to identify individual community nutritional status [14].

Health care professionals play a vital role in promoting healthy eating. Adequate nutrition is essential for the obese and critically ill as well as for those with eating disorders, food allergies, and other clinical problems [15, 16]. However, nutritional care is a complex, multidisciplinary approach that involves physicians, nurses, nutritionists, and other health care providers. Previous studies have documented the role of nursing in nutritional care, particularly for health education, nutrition planning, and guiding patients in their dietary habits [15–17].

Valid and reliable tools are fundamental needs for assessing nutritional self-care among individuals who are at risk of poor health outcomes. The MIN-SC questionnaire was developed in 2005 by Jean Burley Moore and was based on Dorothea Orem's conceptual framework, the Theory of Self-Care Deficit, which has been applied among schoolchildren in the United States, Nicaragua, and Chile [13, 18, 19]. MIN-SC consists of 50 items designed to measure regular dietary intake, planning, and adjustment [19].

Nutritional skills are needed in educational curricula throughout Saudi Arabia. Another critical need is the ability to reliably assess nutritional attitudes, particularly among Saudi adolescents. A tool such as MIN-SC would aid in gathering baseline data for understanding attitudes toward nutritional status and emphasizing the need to design appropriate educational programs. However, MIN-SC has been investigated in different countries and languages with mixed results [20]. For instance, different reliability and validity estimates across countries can affect the significance of comparisons. Furthermore, the psychometric characteristics of the Arabic version of the tool have not been examined in Saudi Arabia. This study was designed to translate and culturally validate MIN-SC among a sample of Saudi students.

## 2. Methods

### 2.1. Design and Setting

This was a descriptive, cross-sectional study of the MIN-SC instrument conducted among college freshman students at King Saud University (KSU) in Riyadh, Saudi Arabia, over a one-month period in February 2019. It was designed to evaluate the psychometric properties of the Arabic version of the questionnaire.

The KSU College of Nursing was established in 1976. It currently offers programs in maternal and child health nursing, medical surgical nursing, community and mental health nursing, and nursing administration and education. The college also offers a master's degree in nursing science. All students who enroll in a nursing program at KSU must first be accepted to the integrated one-year program for health sciences. The criteria for acceptance in the unified program are based on the applicant's scores from a capability exam, cumulative exam, high school, and interview. All students who successfully complete the unified program are directed to one of four health faculties (medicine, dentistry, pharmacy, and applied medical sciences) based on their desire, cumulative average, and program capacity. KSU operates on a single-gender basis.

### 2.2. Population and Sampling

Approximately, 400 students are admitted to the KSU College of Nursing as freshmen, and that number was used to estimate the required sample size. Sample size was calculated using the Raosoft sample size calculator (http://www.raosoft.com/samplesize.html) with a 95% confidence level and a 5% predetermined margin of error. Response distribution for each question was estimated to be 50%, which gave a larger sample size for this research. The calculated sample size was 197, but it was decided that 200 students would be surveyed in an attempt to ensure higher reliability.

### 2.3. Data Collection Procedure

Data were collected by visiting the students at the KSU campus through paper-based questionnaires using convenience sampling. Convenience sampling is a nonprobability sampling technique where study subjects are selected based on certain criteria such as availability at a given time, willingness to participate, easy accessibility, and geographical proximity to the researchers [21].

### 2.4. Data Collection Instrument

MIN-SC was initially developed for use in English and Spanish by Jean Burley Moore et al. in 2005 [19]. The items were developed based on Orem's Self-Care theory, which proposed three domains of self-care (estimative, transitional, and productive). Here, estimative items describe activities related to gathering information and making choices among alternatives (e.g., “I read about nutrition in books”); transitional items describe behaviors designed to plan actions or make decisions (e.g., “I plan my meals so that they are healthy”); and production items describe activities that involve taking actions and evaluating the outcomes (e.g., “I eat breakfast every day”). A follow-up study expanded and validated a newer scale composed of 50 items answered on a five-point Likert-type scale ranging from 1 (never) to 5 (always). This was designed to measure the frequency of nutrition-based behaviors. Higher scores signify healthier child nutrition practices [20].

The contents of the original English instrument were previously validated according to expert opinion, while instrument reliability was established through an alpha coefficient of 0.83 [20, 22]. The translation procedure followed forward-backward translation which was carried out by professional bilingual speakers of English and Arabic, while backward translation was conducted by another set of bilingual professionals. Both versions (the original and back-translated) were adjusted for quality and accuracy by a group of experts. Psychometric properties of the consensus version of the Moore index questionnaire in Arabic were then examined. We evaluated content, face, and construct validity of MIN-SC as follows. Once the tool was prepared in the Arabic language, it was sent to independent reviewers. The reviewers were a senior professor in nursing and an assistant professor and a senior researcher with considerable experience in preparing and designing research questionnaires. Opinions and suggestions about the suitability of the questionnaire were collected from the review team, and changes were made according to the feedback provided by the review team. Face validity of the questionnaire was performed by conducting a pilot study at KSU College of Nursing, under the supervision of the investigator, for the purposes of evaluating the responses of the subjects, measuring the validity of the questionnaire, testing the study tools, and choosing the best methods for data collection and management. The pilot study was completed in one week and involved 67 subjects. After the conclusion of the pilot study, all necessary additions or changes to the study tools were made. The results of the pilot study were not included in the main study.

### 2.5. Data Analysis

Data were analyzed using SPSS, Version 22.0. Exploratory factor analysis (EFA) was applied to determine the factorial structure of MIN-SC. To run factor analysis, we assessed the Kaiser Meyer-Olkin (KMO) and Bartlett's test measures to assess the sample adequacy and sphericity of the Arabic version of MIN-SC, respectively. To explore the structure of survey component, Varimax rotation was used. Items that loaded with eigenvalues >2 were retained in the analysis. Items that loaded with a factor of less than 0.3 were deleted from the analysis. Items that loaded with two or more factors of 0.3 or greater were deleted from the analysis. To keep a factor in the analysis, it must load 3 or more items with no loading on other factors. Items were considered for deletion if their correlation with an item within the same factor was too high (>0.8) or too low (<0.2). The reliability of the Arabic version of MIN-SC was measured through internal consistency using Cronbach's *α* (Cronbach's *α* of ≥0.70 is considered good reliability) [23, 24].

### 2.6. Ethical Considerations

The study was approved by the Institutional Review Board of King Saud University College of Medicine, Saudi Arabia (E-19-3979). All participants provided their written informed consent to participate in this study.

## 3. Results

A total of 200 students were approached during the data collection period. Of those, 60 students (30%) answered incompletely and were therefore excluded from the study. A total of 140 students responded to the Arabic-translated MIN-SC instrument, yielding a response rate of 70%. The content and face validity were established using experts' opinions and students' feedback, respectively. The MIN-SC instrument was shown to be internally consistent with reliable scoring (Cronbach's alpha = 0.910). Exploratory factor analysis resulted in 42 items loading on three main components (estimative, production, and transitional), with a factor loading of eigenvalues > 2. The final model explained 38% of the variance.

As shown in Table 1, Factor 1 contained a total of 18 subscale items. However, Item 49 from Factor 1 is not loaded on the subscale and was deleted from the analysis. Therefore, the final number of items in Factor 1 is 17 and labeled “Productive.” Productive items describe activities that involve taking action and evaluating the outcome (e.g., “I eat breakfast every day”).

Factor 2 contained a total of 17 items. Among those, 2 items were not loaded, or loaded in a factor with less than 0.3, and were deleted from the analysis. The final number of items in Factor 2 is 15 and labeled “Estimative.” Estimative items describe activities related to gathering information and making choices among alternatives (e.g., “I read about nutrition in books”).

Factor 3 contained a total of 12 items. Among those, 2 items were deleted from the analysis. The final number of items in Factor 3 is 10 and labeled “Transitional.” Transitional items describe behaviors related to planning actions and making decisions (e.g., “I plan my meals so that they are healthy”).

We conducted an exploratory factor analysis (principal components analysis) and subsequent Varimax rotation to evaluate construct validity. Kaiser-Meyer-Olkin (KMO) and Bartlett's test indicated that the data were adequate for conducting a principal component analysis (PCA; KMO index = 0.779, *P* < 0.001). The final model retained three factors with eigenvalues >2 and factor loading equal to or greater than 0.43, which explained 38% of the variance. The final validated Arabic MIN-SC contained 42 items loaded among the three components.

### 3.1. Reliability

In terms of internal consistency, Cronbach's alpha scores for the Arabic MIN-SC subscales ranged from 0.831 (Factor 1) to 0.80 (Factor 3). A detail description of factor loading and Cronbach's alpha scores for various subscale of MIN-SC are given in Table 2. A Pearson correlation coefficient was performed to estimate the significance among all items in the nutritional scale. Results indicated that all items were significant at the 0.001 level (Table 3).

## 4. Discussion

This study investigated the psychometric properties of the Arabic version of MIN-SC. Construct validity and PCA revealed a three-component structure (knowledge, estimative, and productive).

This study's reliability coefficient was similar to that of a previous Spanish-language study conducted by Moore [19]. However, it was more reliable than that of an English-language study conducted by Jean Burley Moore et al. in 2005. We believe there are two main reasons for these different MIN-SC results. First, the validity and reliability of MIN-SC in the original study [19] were determined based on a 36-item questionnaire with a smaller sample size, whereas this study used a larger sample size and an expanded 42-item questionnaire. Second, several factors in the Arabic questionnaire were revised, restructured, or removed to obtain a more reliable and valid measurement scale. Variations may also have resulted from cultural or contextual differences.

Spearman and Pearson correlation values for all 42 items were significant at the 0.01 level. In addition, all scaling success rates were excellent based on an assessment of both validities. These results indicate that all questionnaire items represented the underlying construct.

MIN-SC has proven to be a valuable tool in numerous investigations. For instance, it has been used to describe and measure nutritional practices, compare adolescent and parental behavior, examine self-care operations among youth, assess nutritional intake, and determine nutritional effectiveness [19]. At the time of this study, no published research had assessed the psychometric properties of MIN-SC among an Arabic-speaking community. It is with this goal that we translated, designed, and tested the psychometric properties of the Arabic MIN-SC, which was determined to be a valid and reliable tool for assessing nutrition self-care among Saudi students.

The need for this investigation was supported by numerous studies showing that school students do not sufficiently adhere to recommended physical activity and healthy eating habits [25,26]. In one study conducted using a convenience sample of school children to measure self-care, only 2.5% of the children had healthy practices, while 6.9% showed unhealthy behaviors [25]. In 2018, Almutairi et al. conducted a study titled “Health Promoting Lifestyle of University Students in Saudi Arabia.” The study reported that approximately 20% of participants were overweight, while 11.3% were obese [26]. Reports have revealed that a majority of both college and school students do not attend educational programs on health care [20,27].

Studies have shown the importance of nutritional self-care in promoting health quality for both children and adults. Encouraging healthy dietary habits in young children may prevent various chronic health disorders in both childhood and adulthood, including obesity, diabetes, hypertension, cardiovascular disease, cancer, and dental caries [28,29]. Schools and universities may be instrumental in providing information and inculcating students with healthy habits through educational interventions designed to teach proper nutrition. Research has also found that schools and universities are accessible settings for interventions targeted at instilling healthy lifestyle habits among both children and parents [28,29].

The study had some limitations. First, it was conducted at a single institution; therefore, the findings may not be generalizable among other educational settings. However, the Arabic version of MIN-SC demonstrated good psychometric properties and is recommended for use in future studies designed to improve and refine it for greater application among Arabic-speaking communities.

## 5. Conclusion

The Arabic version of the MIN-SC represents a novel scale for the assessment of nutritional intake among adolescents. Unlike other nutritional scales available in other languages, it was designed to include the three main domains (knowledge, production, and estimative). The Arabic version of MIN-SC offers a simple self-care assessment tool that reflects current trends in food and dietary habits and should prove useful to a range of adolescents for achieving adequate health and quality of life.

## Figures and Tables

**Table 1:** Total number of items used in scale.

Factor	Items	No. items	After deletion Cronbach's *a*
F1 production	(5) I learn about healthy food from watching TV	18	17
	(6) I suggest healthy foods for my family to buy	
	(9) I ask my teacher about healthy food to eat	
	(13) I ask my grandparents questions about healthy eating	
	(17) I find out about healthy eating from nurses	
	(19) I study nutrition in school	
	(24) I talk to my friends about which healthy foods to eat	
	(35) I obtain information about nutrition from the Internet	
	(36) I read public announcements about nutritious foods	
	(40) I read about nutritious food to eat in magazines or newspapers	
	(41) I help my family select food to buy	
	(42) I ask other adults questions about healthy eating	
	(43) I eat fruit	
	(44) I eat green vegetables	
	(45) I eat other vegetables	
	(46) I eat meat	
	(47) I drink milk	
	(49) I eat cereal, bread, or tortillas	
F2 production	(2) I read about nutrition in books	17	15
	(4) I study food labels to learn about nutrients in food		
	(8) I try new foods		
	(10) I eat foods containing iron		
	(11) I choose to eat foods that contain vitamins		
	(21) I eat foods that are good sources of vitamin C		
	(18) I make sure the water I drink is clean		
	(22) I wash fruit before eating it		
	(23) I make sure that meat I eat is cooked enough		
	(25) I eat protein at every meal		
	(26) I try to eat food and drink beverages with calcium		
	(27) I eat foods that are good sources of vitamin A		
	(28) I consider whether my meals have enough protein		
	(29) I eat breakfast every day		
	(32) I think about whether what I eat is healthy		
	(34) I choose to eat foods that are low in fats		
	(37) I eat a variety of foods		
F3 transitional			
	(1) I plan my meals so that they are healthy	12	10
	(3) I choose to drink soda instead of water		
	(7) I eat foods that I know are good for me even if I do not like them		
	(14) When I buy a snack I choose a soda rather than fruit		
	(15) I put a lot of salt on the food that I eat		
	(16) I eat the same foods every day		
	(20) I ask my mother which foods are healthy		
	(30) I drink soda instead of fruit juices		
	(31) I would choose to eat sweets instead of a piece of fruit		
	(39) I choose to eat chips and other snacks instead of fruit		
	(48) I eat sweets		
	(50) I eat high-calorie snack foods		
Deleted from PFA			
	(12) If I think I'm gaining too much weight I eat fewer sweets	47	42
	(33) I drink coffee with meals		
	(38) I drink eight glasses of liquid every day		

**Table 2:** Factor loadings (rotated) and Cronbach's alpha for each subscale of nutrition.

Factor	Items		Factor loading	Cronbach's *a*
Production	5	I learn about healthy food from watching TV	0.509	
	6	I suggest healthy foods for my family to buy	0.653	
	9	I ask my teacher about healthy food to eat	0.573	
	13	I ask my grandparents questions about healthy eating	−0.493	
	17	I find out about healthy eating from nurses	0.600	
	19	I study nutrition in school	0.480	
	24	I talk to my friends about which healthy foods to eat	0.619	
	35	I obtain information about nutrition from the Internet	0.528	0.831
	36	I read public announcements about nutritious foods	0.642	
	40	I read about nutritious food to eat in magazines or newspapers	0.469	
	41	I help my family select food to buy	0.540	
	42	I ask other adults questions about healthy eating	0.552	
	43	I eat fruit	0.495	
	44	I eat green vegetables	0.390	
	45	I eat other vegetables	0.376	
	46	I eat meat	−0.485	
	47	I drink milk	0.337	
	49	I eat cereal, bread, or tortillas	0.576	

Estimative	2	I read about nutrition in books	0.506
	4	I study food labels to learn about nutrients in food	0.336
	8	I try new foods	0.578
	10	I eat foods containing iron	0.624
	11	I choose to eat foods that contain vitamins	0.720
	21	I eat foods that are good sources of vitamin C	0.301
	22	I wash fruit before eating it	0.340	0.848
	23	I make sure that meat I eat is cooked enough	0.470	
	25	I eat protein at every meal	0.6.6	
	26	I try to eat food and drink beverages with calcium	0.584	
	27	I eat foods that are good sources of vitamin A	0.617	
	28	I consider whether my meals have enough protein	0.00	
	29	I eat breakfast every day	0.460	
	32	I think about whether what I eat is healthy	0.515	
	34	I choose to eat foods that are low in fats	0.657

Transitional	1	I plan my meals so that they are healthy	0.318
	3	I choose to drink soda instead of water	0.526
	7	I eat foods that I know are good for me even if I do not like them	0.436
	14	When I buy a snack I choose a soda rather than fruit	0.703
	20	I ask my mother which foods are healthy	0.426	0.802
	30	I drink soda instead of fruit juices	0.387	
	31	I would choose to eat sweets instead of a piece of fruit	0.401	
	39	I choose to eat chips and other snacks instead of fruit	0.453	
	48	I eat sweets	0.571	
	50	I eat high-calorie snack foods		

**Table 3:** Spearman correlations between the Moore Index of Nutrition Self-Care scale items.

		Items	Pearson correlation	*P* value
Production	5	I learn about healthy food from watching TV	0.546	≤0.001
	6	I suggest healthy foods for my family to buy	0.648	≤0.001
	9	I ask my teacher about healthy food to eat	0.607	≤0.001
	13	I ask my grandparents questions about healthy eating	−0.314	≤0.001
	17	I find out about healthy eating from nurses	0.678	≤0.001
	19	I study nutrition in school	0.557	≤0.001
	24	I talk to my friends about which healthy foods to eat	0.673	≤0.001
	35	I obtain information about nutrition from the Internet	0.589	≤0.001
	36	I read public announcements about nutritious foods	0.725	≤0.001
	40	I read about nutritious food to eat in magazines or newspapers	0.628	≤0.001
	41	I help my family select food to buy	0.567	≤0.001
	42	I ask other adults questions about healthy eating	0.648	≤0.001
	43	I eat fruit	0.462	≤0.001
	44	I eat green vegetables	0.556	≤0.001
	45	I eat other vegetables	0.501	≤0.001
	46	I eat meat	0.427	≤0.001
	47	I drink milk	0.394	≤0.001
			
	2	I read about nutrition in books	0.578	≤0.001
	4	I study food labels to learn about nutrients in food	0.583	≤0.001
Estimative	8	I try new foods	0.465	≤0.001
	10	I eat foods containing iron	0.593	≤0.001
	11	I choose to eat foods that contain vitamins	0.600	≤0.001
	21	I eat foods that are good sources of vitamin C	0.755	≤0.001
	22	I wash fruit before eating it	0.482	≤0.001
	23	I make sure that meat I eat is cooked enough	0.478	≤0.001
	25	I eat protein at every meal	0.557	≤0.001
	26	I try to eat food and drink beverages with calcium	0.643	≤0.001
	27	I eat foods that are good sources of vitamin A	0.660	≤0.001
	28	I consider whether my meals have enough protein	0.693	≤0.001
	29	I eat breakfast every day	0.340	≤0.001
	32	I think about whether what I eat is healthy	0.538	≤0.001
	34	I choose to eat foods that are low in fats	0.534	≤0.001
			
	1	I plan my meals so that they are healthy	0.597	≤0.001
Transitional	3	I choose to drink soda instead of water	0.549	≤0.001
	7	I eat foods that I know are good for me even if I do not like them	0.646	≤0.001
	14	When I buy a snack I choose a soda rather than fruit	0.717	≤0.001
	15	I put a lot of salt on the food that I eat	0.616	≤0.001
	16	I eat the same foods every day	0.706	≤0.001
	20	I ask my mother which foods are healthy	0.607	≤0.001
	30	I drink soda instead of fruit juices	0.645	≤0.001
	31	I would choose to eat sweets instead of a piece of fruit	0.627	≤0.001
	39	I choose to eat chips and other snacks instead of fruit	0.640	≤0.001
	48	I eat sweets	0.483	≤0.001
	50	I eat high-calorie snack foods	0.447	≤0.001

## Data Availability

The data used to support the findings of this study are available from the corresponding author upon request.
